# Oleh Hornykiewicz, a giant in the understanding and treatment of Parkinson disease

**DOI:** 10.1038/s41531-020-00149-4

**Published:** 2021-01-04

**Authors:** Luigi Zecca, Christian Pifl, Stanley Fahn, David Sulzer, Ruggero G. Fariello

**Affiliations:** 1 Institute of Biomedical Technologies, National Research Council of Italy, Segrate, Milan, Italy; 2 Department of Molecular Neurosciences, Center for Brain Research, Medical University of Vienna, Vienna, Austria; 3 Department of Neurology, Vagelos College of Physicians and Surgeons, Columbia University and the New York Presbyterian Hospital, New York, NY 10032 USA; 4 Departments of Psychiatry, Neurology, Pharmacology, Columbia University Medical Center, New York State Psychiatric Institute, New York, NY 10032 USA; 5 PharmaFox Therapeutics AG, Möhlin, Aargau Switzerland

**Keywords:** Parkinson's disease, Parkinson's disease

## Abstract

Oleh Hornykiewicz (November 17, 1926–May 26, 2020), by demonstrating the loss of dopamine neurons in Parkinson’s disease, introducing the effort to treat the disorder with L-DOPA, and other innovative research, improved the lives of countless individuals and transformed neurology and medical science. Here we celebrate the life and great achievements of an outstanding scientist.

## Hornykiewicz’s youth

**Fig. 1:**
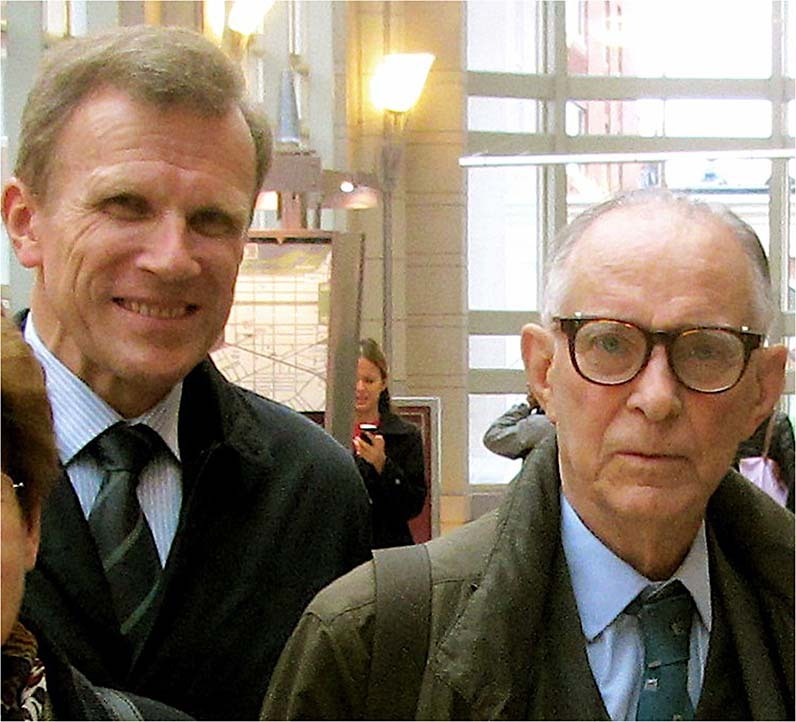
Christian Pifl and Oleh Hornykiewicz. Christian Pifl and Oleh Hornykiewicz, 2014 in Boston on the occasion of the Warren Alpert Foundation Prize ceremony. Photograph by Stephan Hornykewycz.

Oleh Hornykiewicz did not have an easy start in life. He was born in November 1926 in Lviv (Lemberg, Lwow), an area with many nationalities contested over the centuries by Ukraine, Prussia, Lithuania, Sweden, Austria-Hungary, Cossacks, Poland, and Russia. In the beginning of 1940, his family of Ukrainian / Austro-Hungarian origin, moved to Vienna, where he, as a 13 year old immigrant, had to learn German for his schooling. The years in Austria under the Nazi regime and later in post-war years were far from easy. In 1945, after a fierce battle, Vienna was occupied by Allied and Soviet armies and brought to terrible conditions with robberies, revenges, rapes, poverty, and food shortages mainly caused by Soviet army. These experiences produced in the young Oleh a strong character that helped him later to deal with disputes, fights, and frustrations throughout life. At the same time those early difficulties gave him the capacity to fully enjoy the important rewards (Fig. 1) he obtained later on, thanks to his hard work and great ideas.

## Career

Hornykiewicz had contracted tuberculosis, and following the example of his eldest brother who had become a doctor, became interested in medicine, obtaining an MD degree from the University of Vienna in 1951. Fascinated by the burgeoning field of molecular pharmacology, he joined the university’s Pharmacology Department, where he studied the role of the copper-carrying protein ceruloplasmin in another basal ganglia disorder, Wilson’s disease. This was the start of Hornykiewicz’s approach to research, where while trained in pharmacology, he was determined to work with neurologists and neuropathologists, particularly in research using brain autopsy.

Thanks to a British Council Scholarship, Hornykiewicz in 1956–58 joined the lab of Professor Hugh (Hermann) Blaschko’s department in Oxford. Blaschko’s laboratory was then the center for research in catecholamines, particularly due to their characterizations of monoamine oxidase and DOPA decarboxylase.

In Blaschko’s group, he devoted a couple of years to the study of the cardiovascular effects of dopamine. He showed that the effects were due directly to dopamine and not to a metabolite of dopamine.^1^ He also tested L-DOPA and found the same effects.

Hornykiewicz credited Blaschko’s advice to continue to focus on dopamine in his research for his subsequent successful career. Returning to Vienna following reports on dopamine in the brain by Montagu,^2^ Weil-Malherbe and Bone,^3^ and Carlsson et al.^4^ he had read in Oxford, he became a staunch supporter of dopamine’s role as a neurotransmitter. He was particularly impressed by Montagu’s finding that the same compound whose effects he had been studying on blood pressure, was found in the central nervous system, which he described as “bordering, for me, on the sensational”.

Oleh, who was very fair with giving credit to colleagues, was further impressed by the demonstration of dopamine’s high concentration in basal ganglia of healthy human brain^5,6^ and the reversal by exogenous L-DOPA administration of reserpine’s induced akinesia consequent to brain catecholamines depletion.^4,7^

While the loss of the substantia nigra neurons that were pigmented due to the presence of neuromelanin was observed in 1919 by the Russian neuropathologist, Konstantin Tretiakoff, the neurochemical properties of these neurons were unknown. After establishing his laboratory at the University of Vienna, Oleh Hornykiewicz and his young postdoc, Herbert Ehringer, set out to measure brain dopamine and norepinephrine in normal adult human post-mortem brains and in patients who had died of Parkinson disease and postencephalitic parkinsonism. Comparing the concentrations of dopamine and norepinephrine in multiple brain regions, they found a selective dopamine depletion in the caudate nucleus and putamen in patients with both forms of parkinsonism.^8^ Hornykiewicz was also the first to suggest that the loss of these neurons in the midbrain was responsible for the loss of dopamine in the striatum, and in that way was fundamental to introducing the nigrostriatal pathway.^9^

The obvious next step from that discovery was to attempt to treat Parkinson’s patients by administering dopamine’s precursor, levodopa that could reach the brain crossing the blood brain barrier. It took over a year to convince his clinical partner, the neurologist Walther Birkmayer, to attempt intravenous injection of levodopa in patients with Parkinson disease, mostly because Birkmayer was believing that a loss of hypothalamic serotonin, rather than dopamine, was responsible for the disorder. Eventually, they administered small doses of levodopa intravenously to five parkinsonian patients, resulting in the miraculous “awakening” immortalized in the movie now visible at several scientific sites. He succeeded in the beginning of July 1961^10^ and in his words in an interview with Barbara Sommer stated:

“I still remember, of course. I was present at that time in the Lainz hospital and watched the results. It was a spectacular moment to see the patients who could not walk, could not get up from bed, could not stand up when seated, start walking. They all performed these activities like normal. Speech became better. The associated movements, the face expressions; they started laughing and then actually crying with joy. These were patients who could not be helped by any doctor. And then levodopa produced the effects. It was really very spectacular.”

Thanks to that historical event, along with subsequent clinical investigations including the administration of high oral doses of levodopa introduced by Cotzias et al.^11^, millions of parkinsonian patients now enjoy decades of acceptable life in spite of their progressive neurodegenerative condition.

In 1967 Hornykiewicz was offered a professorship at the University of Toronto, Clarke Institute of Psychiatry, where he led the Psychopharmacology Section until he returned to Vienna as Professor of Biochemical Pharmacology in 1976, retaining his scientific commitment in Toronto until the early 1990s. Back in Vienna he was instrumental in the creation of the new Centre for Brain Research at the University.

## Personality

**Fig. 2:**
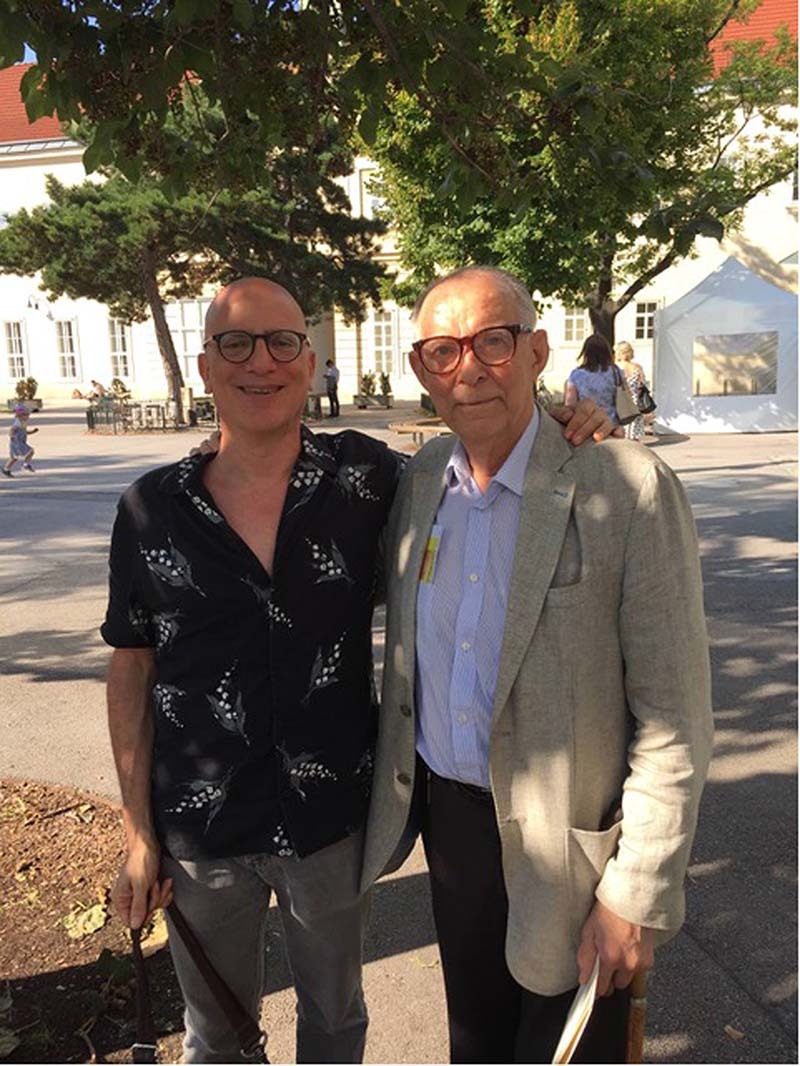
David Sulzer with Oleh Hornykiewicz. David Sulzer with Oleh Hornykiewicz in front of the Narrenturm in Vienna, the earliest building to house psychiatric patients, during the Dopamine 2016 Conference, organized by Harald Sitte and Matthaus Willeit, which featured the Oleh Hornykiewicz 90^th^ birthday symposium. Photograph by Luigi Zecca.

Oleh Hornykiewicz was a gentleman and a family man. With his wife, Christine, they had Maria, Nikolai, Stephan, and Joseph and together enjoyed an intense family life until Christine’s passing away in 2017. Hornykiewicz was an “independent thinker” like most ingenious people. Obviously, he was open minded and listened to all scientific opinions, but at the end he drew his personal conclusions and road maps for the experimental paths that led him to great achievements. Although exposed to different mentors whom he respected highly, he was not a follower of anyone. Hornykiewicz’s work was novel and centered on human brain disease. Sometimes having one prevailing mentor induces people to remain intellectually pupils for their entire life. When meeting somebody for the first time, within the blink of an eye, he immediately understood who you were, your standing, and your potential. Even though he was a highly respected and recognized scientist, he always remained very open to listening and discussing opinions with anybody, without arrogance (Fig. 2). He was not politically correct, but instead he was realistically direct.

## Scientific controversies

Hornykiewicz endured multiple hard and long-lasting controversies during his career, but by using simple and accurate approaches, was eventually acknowledged by the field to have made fundamental discoveries. Having published his two seminal papers in German language journals it was a challenge for him to convince influential members of the international neuroscience community about the pivotal role of dopamine in motor behavior, and about its absence in the neostriatum being the cause for PD symptoms.^9^ This was in part because some eminent clinical scientists claimed a primary role of serotonin and noradrenaline in the motor symptoms of parkinsonian patients, and stated that L-DOPA could not be an effective treatment for Parkinson disease, and would not be superior to the anticholinergic medications then in widespread use.^12^

## Disputes and troubles

His fiercest and most unfortunate dispute was with Walther Birkmayer regarding the paternity of the idea for L-DOPA replacement therapy. As shown in Hornykiewicz’s early studies and discussions, he had developed this idea in a total autonomous way before treating the first patients. It took Hornykiewicz about a year to convince Birkmayer to try L-DOPA on patients, although Birkmayer and colleagues tried to communicate a different history. This dispute became a bitter split in Vienna’s neuroscience community that spilled way beyond Austria’s borders. The publication of stories attributing to others the priority of L-DOPA treatment was but one of the many attempts to undermine Hornykiewicz’s achievements.

It is likely that the acrimony and virulence of these envy-driven attacks played a role in the failure to recognize his accomplishments with the Nobel Prize in the year 2000. The omission of Hornykiewicz was widely considered to be a grave error and injustice, made more so given that the mention of the treatment of Parkinson disease was a stated motivation for the Nobel award. A letter from the international neuroscience community, notably not instigated by Hornykiewicz himself but promoted by Ali Rajput,^13^ complaining about the blatant oversight by the Nobel committee was signed by 250 scientists.

## Heritage

Oleh Hornykiewicz taught us about science and life. In science, his name and his work will go down in history as the one who first provided the pathophysiological explanation for loss of motor function in Parkinson’s disease, introducing an effective, albeit symptomatic, treatment for a major neurodegenerative disorder.

Hornykiewicz stated that “I felt the best thing was to go directly to the human brain and see whether there was a change in Parkinson’s Disease or not” (in his speech to the award of honorary membership of the Austrian Pharmacological Society 2003). He demonstrated that the post-mortem biochemical analysis of nervous tissue provides reliable and relevant clues to the pathogenesis “in vita” of diseases, giving birth to the field of “pathobiochemistry” that has led us to the contemporary understanding of many neuropsychiatric disorders.

**Fig. 3:**
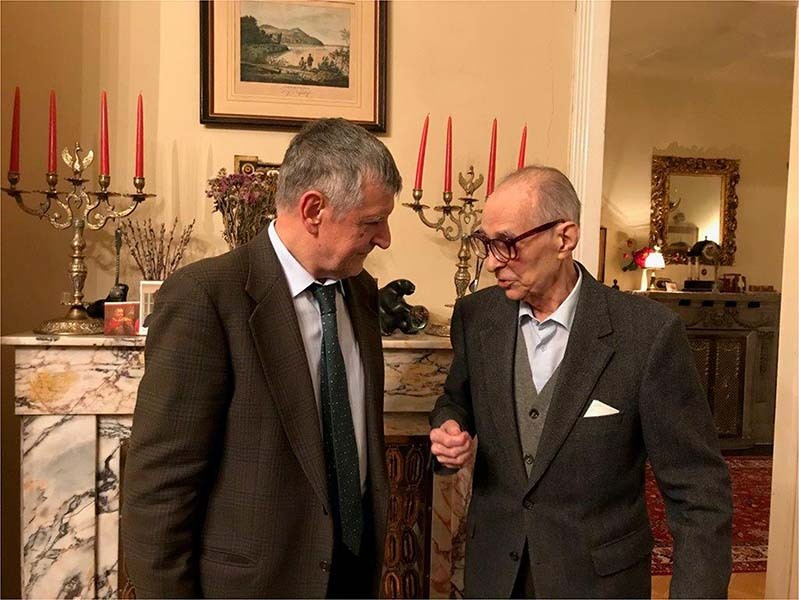
Luigi Zecca and Oleh Hornykiewicz. Luigi Zecca discussing with Oleh Hornykiewicz in his house in Vienna, the 3^rd^ December 2019. Photograph by Christian Pifl.

He provided an example of how it is important for scientists to have original, independent ideas rather than follow the fads of the moment, and how once those ideas are corroborated by solid experimental evidence, to be brave enough to carry them forward. That means we have to be ready to fight for our ideas, but this is obviously not a life for all humans. His driving force was scientific curiosity and not a wish for power and career, unlike what is commonly sought by many. Even in the final days of his life, talking with him you could feel his excitement and intellectual curiosity to understand even the minutiae of science and life (Fig. 3). He suggested simple ideas that generated extraordinary results. In the scientific approach, he was “translational” long before the introduction of this term. He demonstrated the importance of studying brain diseases using human brain samples from autopsy and emphasized that we need to keep in mind the limitations of cellular and animal models. The important discovery of the loss of dopamine in PD was not published in a major journal at that time, and even now such an “observational” discovery submitted to a highly ranking journal would typically be rejected, as it would be absent from these models and does not require analysis by high-tech digital approaches.

The methods he used were quite simple but straightforward and accurately carried out. In life, Oleh showed how undergoing difficult situations in youth may help people to grow strong and ready to face and solve problems in the rest of their life. He also provided an example of how a stable, loving family could provide support during a challenging scientific career.
